# A Novel Two-Tag System for Monitoring Transport and Cleavage through the Classical Secretory Pathway – Adaptation to HIV Envelope Processing

**DOI:** 10.1371/journal.pone.0068835

**Published:** 2013-06-19

**Authors:** Zachary D. Stolp, Aleksandr Stotland, Samantha Diaz, Brett J. Hilton, Wesley Burford, Roland Wolkowicz

**Affiliations:** Department of Biology, San Diego State University, San Diego, California, United States of America

## Abstract

The classical secretory pathway is essential for the transport of a host of proteins to the cell surface and/or extracellular matrix. While the pathway is well-established, many factors still remain to be elucidated. One of the most relevant biological processes that occur during transport involves the cleavage of pro-proteins by enzymes residing in the endoplasmic reticulum/Golgi/TransGolgi Network compartment. Teasing out the requirements involved in the classical secretory pathway and cleavage during transport would shed new light into mis-regulation leading to disease. Current methodologies fail to link transport and cleavage at the single cell level. Here, we describe a cell-based assay that relies on an engineered protein scaffold that can discriminate between transport to the cell surface, in the absence or presence of cleavage. Our novel two-tag system works in a robust and quantitative manner and distinguishes between cleaved and non-cleaved events based on cell surface expression of one or two epitope tags, respectively. Here, we have used the HIV-1 envelope as a substrate, which is cleaved during transport, as proof of principle. Importantly, this assay can be easily coupled to existing siRNA-based screens to identify novel regulators and effectors involved in transport and/or cleavage of cell surface proteins. In addition, unlike other *in vivo* based assays, the assay described here can also be easily adapted to drug discovery purposes.

## Introduction

The classical secretory pathway is critical for normal cell function as it is utilized for the transport of many proteins to the cell membrane or for their secretion into the extracellular matrix (ECM). In order to reach the secretory pathway, proteins are targeted to the Endoplasmic Reticulum (ER), and travel through the Golgi and Trans-Golgi Network (TGN) where they can be co- and post-translationally modified into mature forms prior to their final destination; insertion within cellular membranes or secretion [1]. Modifications to the proteins include glycosylation, myristoylation, palmitoylation, and importantly, processing of protein precursors into mature forms through proteolysis [1–4]. Proteolytic processing within the secretory pathway relies on a wide array of ER/Golgi/TGN resident cellular proteases, including signal peptidases (SP), signal peptide peptidases (SPP), and proprotein convertases (PC) [5–7]. While SPs and SPPs remove signal peptides/sequences required for the targeting of proteins to the ER and secretory pathway, PCs cleave proteins or remove inhibitory domains leading to conformational changes and the activation of proteins into their mature forms [1,5,6]. ER/Golgi/TGN resident proteases play an essential role in normal function, and mis-regulation of these enzymes has been attributed to the development of cancer, Alzheimer’s, and other diseases [8,9].

Proteolytic processing of proteins within the vesicles of the secretory pathway is further exploited by many pathogens; protozoan, bacterial and viral, entering or exiting the cell. Furin, the first discovered and best characterized member of the PC family, has been shown to be required for the propagation of many human viral pathogens [6,8,10–12]. These include the Human Immunodeficiency Virus Type 1 (HIV-1), and some members of the viral *Flaviviridae* family such as Dengue virus and tick-borne encephalitis virus, among others [2,12–14]. Furin processing of the HIV-1 envelope (Env) represents a good example of substrate recognition/proteolytic activity during transport through the classical secretory pathway. Furin substrates are relatively conserved and contain a structural motif consisting of a core domain flanked by two flexible polar regions [6,15,16]. The core region contains the canonical highly basic amino acid recognition sequence R-X-K/R-R which fits within the catalytic domain of Furin and determines its binding affinity [15–17]. The two flexible regions facilitate access to the core domain, suggesting that proper secondary and tertiary structures are necessary for exposing the core recognition site for Furin-based processing [15,16].

As part of the HIV-1 life cycle, the viral Env glycoprotein travels through the secretory pathway and is embedded within the cellular membrane where it becomes part of the viral enveloped particle upon budding [2]. Env is initially translated into the polyprotein precursor gp160 protein, a well-established Furin substrate. Furthermore, the processing of gp160 is critical for the production of infectious HIV viral particles [2,12,18,19]. As a trimer, Env is post-translationally cleaved by Furin into gp120 and gp41, which remain non-covalently associated [20,21]. In the viral progeny, gp120 mediates binding to the primary CD4 host receptor and subsequent binding to CXCR4 or CCR5 co-receptors [18,22,23]. Receptor binding of gp120 causes a conformational change which exposes the fusion peptide of gp41 necessary for fusion with the cell membrane, and allows entry of the viral particle into the cell [23]. Thus, blockade of Env processing by Furin, while not hindering viral particle release, does result in non-fusogenic virions [19,24,25]. Therefore, inhibiting the process of gp160 recognition and/or cleavage represents an attractive novel approach for the discovery of new HIV antivirals. However, as Furin, enriched in the TGN, is an essential cellular protease, is not a viable target and competitors of HIV Env recognition/cleavage rather than Furin inhibitors, are thus needed [6,26]. The impact of HIV on human health, together with the lack of drugs targeting the processing of HIV Env and the well-characterized recognition/cleavage site of Furin and/or similar PCs, reinforces it as an ideal substrate, and for these reasons we have used it here as proof of concept of our novel cell-based assay system.

Previously, the discovery of Furin competitors has been hampered by the limited tools available. Common methodologies to detect processing/cleavage by PCs involve time-consuming techniques such as Western blotting for assessment of cleavage products [27]. Current high-throughput assays rely on fluorogenic substrates and do not represent the natural cellular *milieu* [28]. Several biochemical assays, while high throughput, are not only not performed in the cellular context but are not in the proper cellular compartment, i.e the ER/Golgi/TGN. Moreover, non-cellular assays cannot account for cytotoxic effects of drugs under development. While addressing many of these issues, a cell based assay that relies on secreted alkaline phosphatase, while invaluable for drug discovery, does not monitor enzymatic activity on a single cell level and does not couple cell with phenotype [29]. Thus this assay does not allow for pinpointing cells within a population, which is crucial for the screening of endogenously expressed targeted peptide libraries or cells expressing cDNA or siRNA libraries [30,31]. Other assays include the one described by Hobson et al., which while cell-based, is limited to cell surface cleavage and relies on a rather complex design [32].

Here, we present a novel *in vivo* cell-based assay that quantitatively monitors processing and/or transport to the cell surface at the single cell level. As proof of principle, we adapted our assay system to the HIV Env’s gp120/gp41 boundary cleaved by Furin and similar PCs. The assay is based on an engineered scaffold that utilizes the classical secretion transport pathway to travel to the cell surface. As such, it is the perfect tool to monitor cleavage events that are known to occur in the ER/Golgi/TGN compartment as well as the process of transport to the cell surface itself. Our assay thus combines cleavage and transport, two biological processes that occur in the ER/Golgi/TGN compartment. The assay relies on a two-tag based method that enables, in an elegant, robust and straightforward manner, to distinguish between cleaved and non-cleaved events that occur in the ER/Golgi/TGN.

## Results

### *The assay*

**Figure 1:**
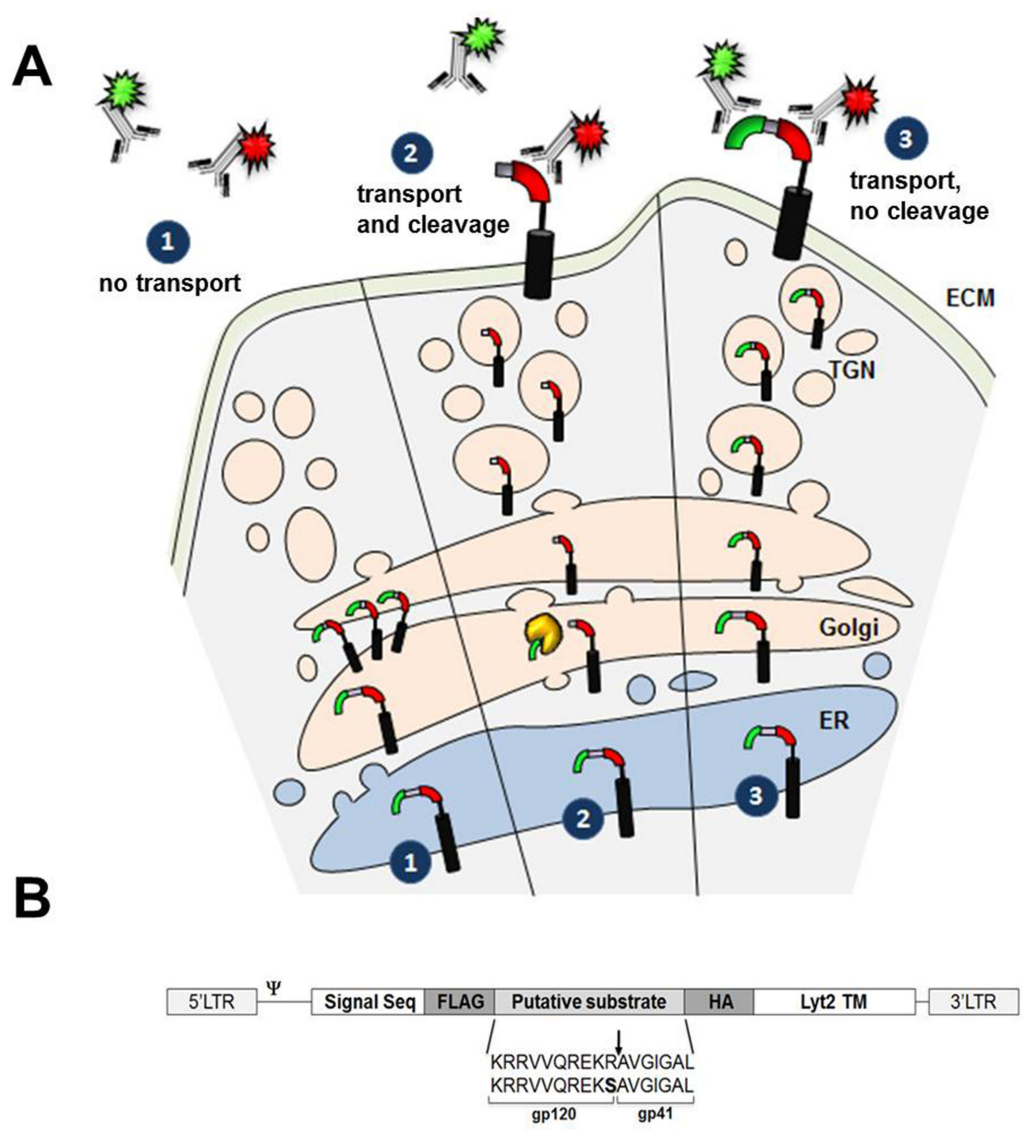
Depiction of the assay. A. Process in the event of lack of transport. No tag will be detected on the cell surface (*Pathway 1*). Process in the event of transport and cleavage. Only one tag can be detected on the cell surface (*Pathway 2*). Process in the event of transport but lack of cleavage. Two tags are detected on the cell surface (*Pathway 3*). The cell surface tag expression can be detected through fluorescent-coupled antibody recognition. Red and green tag represent HA and FLAG tags, respectively. ER: Endoplasmic Reticulum, TGN: TransGolgi Network, ECM: Extracellular Matrix. B, Retroviral construct utilized for the assay. 17 amino-acids of the wild-type gp120/gp41 boundary (*upper*) and mutant (*lower*) sequences used are represented as substrates. R-to-S substitution is represented in bold. Signal Seq: Prolactin signal sequence, LTR: Long terminal repeat, TM: transmembrane domain, ψ: packaging signal.

Our assay relies on a double-tagged scaffold that is used to discriminate between cleaved and non-cleaved events that occur in the ER/Golgi/TGN compartment during transport to the cell surface. Discrimination is based on the cell surface expression of one or two tags, respectively. The scaffold mimics the natural process of a protein traveling through the classical secretory pathway to the cell surface. In the event of recognition and cleavage by proteolytic enzymes residing in the ER/Golgi/TGN compartment, one tag is detected. Conversely, if the process is blocked or hindered both tags are retained and present on the cell surface. A depiction of the assay is shown in Figure 1A. Tags can be recognized by fluorescence-coupled antibody staining and analyzed through classical flow cytometry, or alternatively, through fluorescence-based imaging techniques.

The assay relies on the expression of an engineered protein scaffold that travels to the cell surface. The scaffold contains the prolactin signal sequence at its N’ terminus to ensure that it is targeted to the ER, the first step required for transport through the classical secretory pathway. Downstream of the signal sequence we introduced a cassette containing the FLAG and HA tags flanking a putative protease substrate. Finally, the transmembrane domain (TM) of the murine CD8a glycoprotein receptor homolog Lyt2 was added at the C-terminal tail of the fusion. A previously engineered Lyt2 TM tagged with the FLAG epitope was demonstrated to travel to the cell surface (data not shown), providing the appropriate scaffold for the assay. A four glycine linker was introduced between the HA tag and TM to increase flexibility. The engineering of the scaffold, in such a way, ensures proper localization of the substrate within the luminal face of the ER/Golgi/TGN, as it travels through the secretory pathway. If transport is jeopardized, no tags will be detected on the cell surface (Figure 1A, pathway *1*). In the event of cleavage the FLAG-tag is released and only the HA tag can be detected on the cell surface (Figure 1A, pathway *2*). Conversely, if protease recognition/cleavage is blocked the FLAG-tag is retained and both tags travel to the surface (Figure 1A, pathway *3*).

### *Robust distinction between cleaved and non-cleaved HIV gp120/gp41boundary by flow cytometry*

17 amino-acids comprising the gp120/gp41 boundary of the HXB2 HIV-1 T-tropic prototypic strain was used as substrate to corroborate the utility of the assay, and thus serves as proof of principle. The Env boundary, which is known to be cleaved by Furin and similar PCs, was inserted in-frame between the FLAG and HA-tags (Figure 1B).

**Figure 2:**
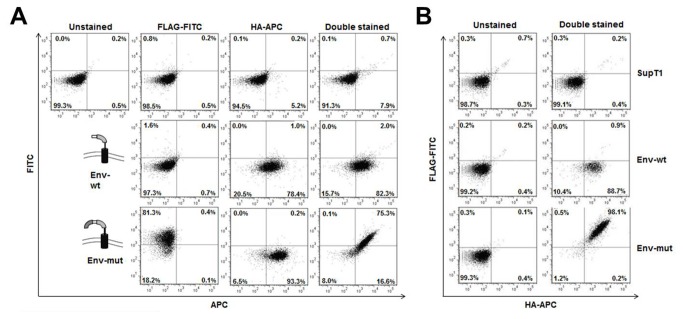
Flow cytometry analysis of cell lines expressing the assay. A. Naïve HEK293T cells or HEK293T clones expressing Env-wt or Env-mut were stained with FLAG-FITC, HA-APC or both antibodies and analyzed by flow cytometry. B. Naïve SupT1 T-cells or SupT1 clones expressing Env-wt or Env-mut were doubled-stained with FLAG-FITC and HA-APC antibodies and analyzed by flow cytometry.

The Env boundary-bearing scaffold construct was introduced into a Murine Leukemia Virus-based retroviral vector used in consequent experiments for stable expression in mammalian cells [31]. A known non-cleavable recognition sequence containing an Arg-to-Ser mutation was used as control [33]. It is important to mention that this substitution serves as control for the assay and not to prove Furin specificity, which is beyond the goal of the assay. Wild-type and mutant boundaries are referred to as Env-wt and Env-mut, respectively (Figure 1B). Retrovirally transduced adherent HEK293T cells were analyzed by flow cytometry and sorted based on HA cell surface expression, ensuring that proper transport to the cell surface occurred within the purified population. This population was clonally sorted into 96-well plates and amplified to create assay-expressing cell lines. HEK293T clones were then analyzed by flow cytometry following staining with anti-HA, anti-FLAG or both antibodies. A secondary allophycocyanin (APC)-coupled antibody was used to detect HA (HA-APC) and a fluorescein isothiocyanate (FITC)-coupled antibody was used to detect FLAG (FLAG-FITC). The analysis showed a dramatic distinction between Env-wt and Env-mut expressing cells. While HA-APC staining was robust with both cell lines (78-93%), it was FLAG-FITC positive only with Env-mut (81% versus 1%). This result was further corroborated by double staining, with 2% of cells co-expressing FLAG and HA in Env-wt cells in contrast to 75% in Env-mut cells (Figure 2A). Complete abrogation of the Env-mut boundary cleavage nicely corroborates that cleaved and non-cleaved events can be discriminated based on FLAG surface expression.

A similar result was obtained with non-adherent SupT1 T-cell line clones, chosen as a cell type that better mimics the natural *milieu* of HIV infection. While 98% of Env-mut cells stained for both FLAG and HA tags, only 0.9% Env-wt cells were double-positive. Importantly, while Env-wt clones lost most of FLAG surface expression, both Env-wt and Env-mut were positive for HA (88-98%) (Figure 2B), demonstrating that both scaffolds travel to the cell surface, regardless of proteolytic processing.

### *Rescue of FLAG surface expression upon inhibition of cleavage as analyzed by flow cytometry*

**Figure 3:**
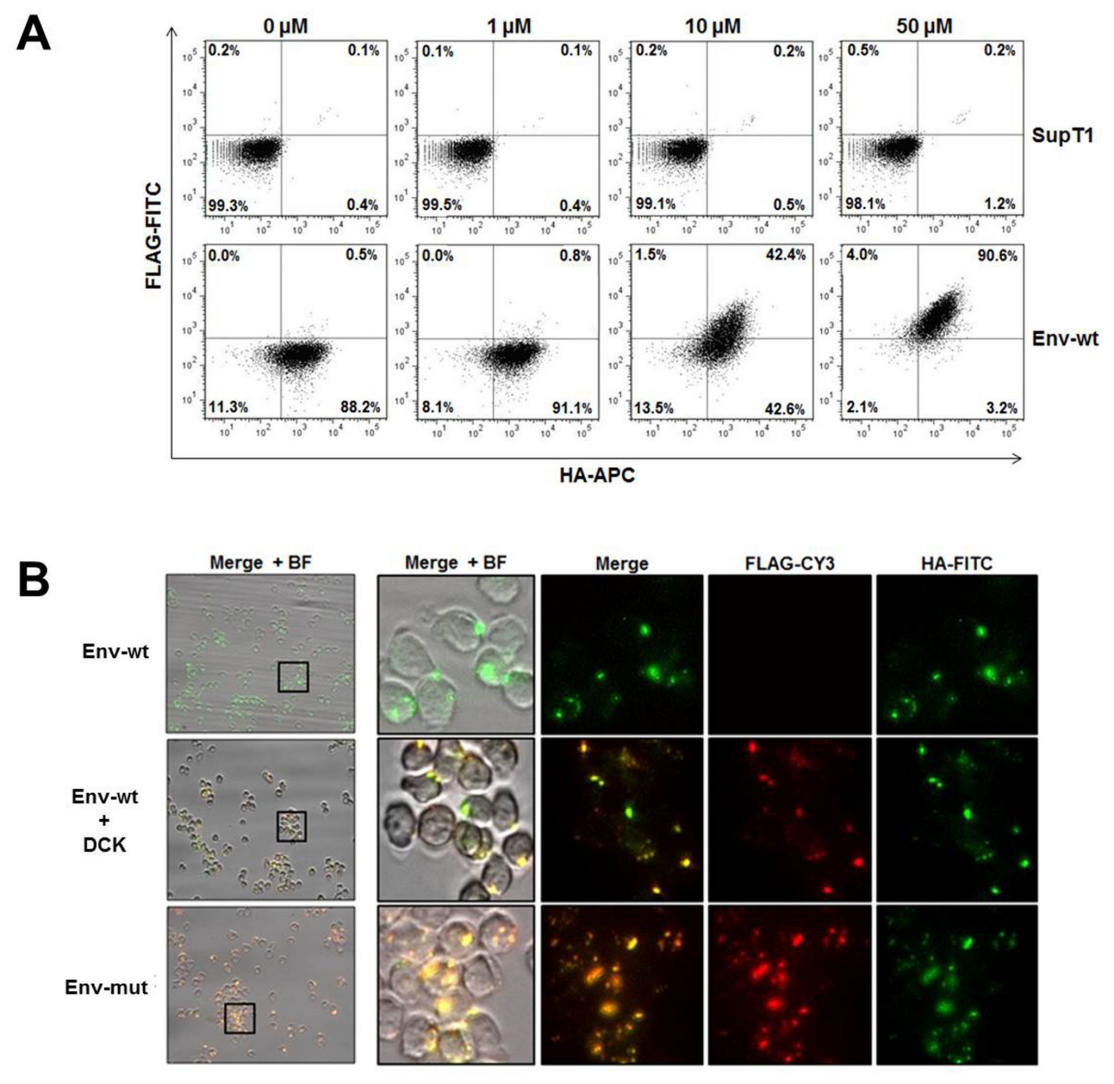
Inhibition of Furin-dependent cleavage reconstitutes FLAG surface expression. A. SupT1 clones expressing Env-wt were treated with increasing concentration of the DCK Furin inhibitor, were doubled-stained with FLAG-FITC and HA-APC antibodies and analyzed by flow cytometry. B. Fluorescent microscopy images of SupT1 clones expressing Env-wt with and without DCK, and Env-mut, stained with FLAG-CY3, and HA-FITC. Single channels, merge and bright field (BF) are shown for comparison. DCK: decanoyl RVKR chloromethyl ketone.

In an attempt to further demonstrate the robustness of the assay it was important to prove whether a Furin/PC protease inhibitor can reverse the observed trend. For that purpose, SupT1 clones expressing Env-wt were analyzed by flow cytometry following incubation with increasing concentrations of the Furin inhibitor decanoyl RVKR chloromethyl ketone (DCK), known to bind to the Furin catalytic site and block its activity [27]. FLAG surface expression is progressively recovered in the presence of 1 µM, 10 µM and 50 µM DCK for 48 hours at 37^°^C, increasing from 0.8% at 1 µM to 42% and 90% respectively (Figure 3A). Control DMSO treated cells showed no FLAG surface expression (data not shown). These results proved the assay to be robust and enable clear discrimination between cleaved and non-cleaved events.

### *Fluorescent microscopy corroborates the robustness of the assay*

To further corroborate the robustness of the assay and utility to imaging-based plate readers, we analyzed the assay by microscopy. For that purpose, tag surface expression of the same clones was further analyzed through fluorescence microscopy prior to and following incubation with 50 µM DCK, Env-mut was used as control. As seen in Figure 3B, Env-mut clones are both green (HA-FITC staining) and red (FLAG-Cy3 staining), while clones expressing Env-wt are only green, corroborating the flow data. Importantly, when treated with DCK, FLAG staining is restored as detected by red fluorescence. These results confirm that the assay can be utilized for imaging-based techniques as well as flow cytometry.

## Discussion

We have developed a novel *in vivo* cell-based assay that discriminates between cleaved and non-cleaved events that occur in the ER/Golgi/TGN apparatus. The engineering of a scaffold protein that utilizes the classical secretion transport and travels to the cell surface was proven to be the perfect tool to monitor transport and cleavage events known to occur in the ER/Golgi/TGN compartment. The cleavage of the minimal gp160/gp41 HIV-1 envelope boundary (Env-wt) between the HA and FLAG tags was used to validate the efficacy of our cell based assay and was readily assessed in a very robust and straightforward manner. A single Arg-to-Ser point mutation within the gp160/gp41 HIV-1 boundary (Env-mut) completely abrogated cleavage, as demonstrated by a strong FLAG surface expression. The robustness of the assay was further demonstrated with Env-wt, where a complete loss of FLAG surface expression was observed. Importantly, in the presence of the known Furin/PC protease inhibitor DCK, a total reconstitution of FLAG surface expression was observed, corroborating that the loss of FLAG was specifically due to the cleavage of the substrate by Furin/PCs while traveling to the cell surface. While the minimal Env boundary is not intended to mimic the complex three dimensional and trimerization requirements of the HIV Env that occur *in vivo*, it was intended to prove the utility of the assay. Our results clearly demonstrate that the assay permits the assessment of Furin-mediated processing. While the assay does not address enzyme specificity and it is intended to monitor cleavage in a robust manner, it does suggest Furin-based processing, as shown by the complete abrogation of cleavage in the presence of a single point mutation that disrupts the well-conserved Furin recognition/cleavage site (see Table 1). Nonetheless, enzyme specificity should be easily investigated by utilizing the assay in conjunction with RNA interference-based technologies or knockout cell-lines.

**Table 1:** Known Furin substrates and their origin.

**Substrate**	**Function**	**Sequence**	
**Viral**			
**HIV-1 gp160**	**Envelope/viral entry**	PTKAKRRVVQREK**R**	AVGIGA
**HIV-2 gp140**	**Envelope/viral entry**	PVKRYSSAPVRNK**R**	GVFVLG
**Dengue Virus pr-M**	**Envelope/viral entry**	GTCTTTGEHRREK**R**	SVALVP
**Influenza A Virus HA**	**Envelope/viral entry**	ATGPRNVPQRRKK**R**	GLFGAK
**Human Papilloma Virus 16 L2**	**Minor Capsid**	MRHKRSAKRTK**R**	ASATQL
**Ebola Virus (Zaire) GP**	**Envelope/viral entry**	GVAGLITGGRRTR**R**	EAIVNA
**Bacterial**			
** *Shigella* *dysentariae* Shiga Toxin subunit A**	**Toxin Activation**	LILNCHHHASRVA**R**	MASDEF
***Bacillus anthracis* Anthrax Toxin Protective Antigen**	**Toxin Activation**	PELKQKSSNSRKK**R**	STSAGP
***Corynebacterium diphtheriae* Diptheria Toxin**	**Toxin Activation**	YMAQACAGNRVR**R**	SVGSSL
** *Pseudomonaaeroginosa* exotoxin A**	**Toxin Activation**	HLPLETFTRHRQP**R**	GWEQLE
**Eukaryotic**			
**Human β Amyloid Precursor Cleaving Enzyme 1 (BACE1)**	**Enzyme Processing**	SGLGGAPLGLRLP**R**	ETDEEP
**Human Matrix Metalloproteinase 2 (MMP2)**	**Structural**	DLDQNTIETMRKP**R**	CGNPDV
**Human Insulin-like Growth Factor 1 (IGF1)**	**Signaling**	LKPAKSARSVRAQ**R**	HTDMPK
**Human Transforming Growth Factor β (TGFβ)**	**Signaling**	YRLESQQTNRRKK**R**	ALDAAY
**Human Vascular Endothelial Growth Factor C (VEGF C)**	**Signaling**	KLDVYRQVHSIIR**R**	SLPATL

Ref [9,13,15,16,33].

### *Adaptability to other substrates*

While the HIV envelope gp120/gp41 boundary is a well-recognized target of Furin/PCs, it was chosen as proof of principle in part due to the impact of HIV-1/AIDS. The simplicity of the assay allows for its adaptation to any other substrate of choice, provided cleavage occurs within the ER/Golgi/TGN compartment. The assay can thus provide a platform for monitoring the cleavage of proteins from viral, bacterial or eukaryotic origin, processed and/or cleaved during transport from or residency within the ER/Golgi/TGN compartment. Table 1 shows, as example, some of the known Furin substrates that could be studied with our assay. However, this assay system targets all the enzymes that cleave within the luminal face of the ER/Golgi/TGN compartment, including non-Furin PC and SP family members, or the β-site amyloid precursor protein cleaving enzyme (β-secretase), critical for the onset of Alzheimer’s disease [7,9,16].

### *Assessment of transport through the classical secretory pathway*

In the assay the HA tag is used to evaluate proper transport to the cell surface, while the FLAG tag is used to evaluate cleavage. As such, while we focused on the presence or absence of FLAG on the cell surface as a correlate of HIV Env cleavage, the assay could also focus on the absence or presence of the HA tag for the analysis of factors strictly required for transport or affecting the secretory process with no role in proteolytic processing. Thus, our assay can be used in conjunction with siRNA-based screens to identify regulators and effectors involved in transport.

### *Adaptability to other tags*

The assay is based on the HA and FLAG independent tags, chosen for the availability of widely utilized, affordable fluorescent-coupled antibodies. The assay should be adaptable to any tag or fluorescent protein of choice, provided they are recognizable on the cell surface by flow cytometry or microscope imaging-based techniques and can be engineered to flank both sides of a putative substrate. For instance, a fluorescent protein such as green fluorescent protein (GFP) could substitute for the FLAG tag, increasing versatility of the assay for flow-cytometry-microscopy imaging-based coupled technologies, where GFP localization would serve as biosensor for processing. Similarly, we have previously reported an *in vivo* cell based assay for HIV protease activity based on the inducible activation of a GFP expression cassette in cell nuclei [34,35].

### *Utility for drug discovery*

The assay represents an *in vivo* cell-based platform for drug discovery as it can be exploited for the screen of inhibitors/competitors against the process of cleavage of any substrate of interest and/or against the activity of the enzymes and their co-factors responsible for cleavage. The robustness and simplicity of the assay is an asset for drug discovery screening. Our cell-based platform can utilize flow cytometry and/or microscopy to assess the activity of proteolytic enzymes that reside or function in the secretory pathway. As the phenotypic outcome is coupled to the cell, the assay allows for the sorting and amplification of single cells, and as such the assay is distinguishable from other existing assays. The coupling of the phenotype to the individual cell, in contrast to assays that rely on secretion of enzymatic/fluorogenic substrates, can be exploited to screen endogenously expressed/targeted peptide libraries or cells harboring cDNA or siRNA libraries. Naturally, the assay allows for the screening of chemical compound and/or combinatorial libraries as well. The robust and quantitative results obtained with the HIV-1 Env boundary make the assay an attractive method for the screen of Furin competitors against Env recognition and/or cleavage to discover novel anti-viral compounds targeted against HIV. Moreover, as the versatility of the assay allows the replacement of the chosen substrate, the assay can be used for drug discovery against a wide array of targets involved in diseases that rely on the secretory pathway for transport and/or cleavage.

In conclusion, our versatile and robust *in vivo* cell-based assay combines both cleavage and transport. It can thus be exploited to study the proteins and factors that either affect or are required for protein cleavage/processing/maturation in the ER/Golgi/TGN compartment. On the other hand, as the assay relies on a scaffold protein that travels to the cell surface, it can be used to learn about the proteins and factors that are required for transport to the cell surface.

## Materials and Methods

### *Cells*

Human T-cell line SupT1 was obtained from the American Type Culture Collection (ATCC, Manassas, VA). Cells were maintained in complete RPMI 1640 media supplemented with 10% fetal bovine serum (Gemini Bio-Products, West Sacramento, CA), glutamine (2 mM), penicillin G (100 units/mL), and streptomycin (100 µg/mL). Phoenix GP and HEK293T cell-lines (Nolan Lab, Stanford University, CA) were maintained in Dulbecco’s Modified Eagle’s media supplemented with 10% fetal bovine serum (Gemini Bio-Products, West Sacramento, CA), glutamine (2 mM), penicillin G (100 units/mL), and streptomycin (100 µg/mL).

### *Antibodies and reagents*

Anti-FLAG antibody was obtained from Sigma Aldrich (St. Louis, MO). Anti-HA, anti-mouse IgG Alexa Fluor 488, anti-rabbit IgG Alexa Fluor 647, anti-mouse IgG Alexa Fluor 555 and anti-rabbit Alexa Fluor 488 used for fluorescent microscopy were obtained from Cell Signaling (Beverly, MA).

### *Plasmids*

The construct pBluescript.FLAG-HIV-1MinWT.i.Zeocin was created by digesting the previously constructed pBMN.FLAG-SBP-Citrine-Lyt2.i.Blasticidin, which contains a Kozak sequence and Prolactin signal sequence with XhoI/NotI restriction enzymes and ligating the resulting FLAG-SBP-Citrine fragment into pBluescript SK+ (Stratagene, Santa Clara, CA) digested with XhoI/NotI. The HIV-1MinWT sequence was amplified from the HXB2 strain of HIV-1 with the forward primer TATATAAAGCTTAAGAGAAGAGTGGTGCAGAGAGAAAAA, which contains a HindIII site, and reverse primer AGAGCAGTGGGAATAGGAGCTTTGGAATTCTATATA, which contains an EcoRI site. The product was ligated into pBluescript.FLAG-SBP-Citrine-Lyt2 digested with HindIII/EcoRI restriction enzymes to create pBluescript.FLAG-HIV-1MinWT-Lyt2. The vector pBluescript.FLAG-HIV-1MinWT-HA-Lyt2 was created by amplifying the HA tag using the forward primer TATATAGAATTCTACCCATACGATGTTCCAGATTACGCT, which contains an EcoR1 site, and the reverse primer TACGATGTTCCAGATTACGCTAGATCTGGTGGCGGAGGGCAATTGTATA, which contains a BglII site, Glycine Linker, and MfeI site. The PCR product was ligated into pBluescript.FLAG-HIV-1MinWT-Lyt2, utilizing the EcoRI site. The pBluescript.FLAG-HIV-1MinMut-Lyt2-HA-Lyt2 vector was created using the forward primer TATATAAAGCTTAAGAGAAGAGTGGTGCAGAGAGAAAAAAGCGCAGTGGG, containing a HindIII site, which overlaps with the reverse primer CAGAGAGAAAAAAGCGCAGTGGGAATAGGAGCTTTGGAATTCTATATA, containing an EcoRI site. The product was ligated into pBluescript.FLAG-HIV-1MinWT-Lyt2 digested with HindIII/EcoRI restriction sites. The plasmids pBMN.FLAG-HIV-1MinWT-Lyt2.i.Zeocin and pBMN.FLAG-HIV-1MinMut-Lyt2.i.Zeocin, were created by digesting the pBluescript versions of the vectors with XhoI/NotI restriction enzymes and ligating the product into retroviral transfer vector pBMN.i.Zeocin utilizing the XhoI/NotI restriction sites.

### *Virus production and transductions*

For the production of MLV based virus, a 10 cm^2^ plate of Phoenix GP cells at 50% confluence was transfected with 3 µg of the packaging vector (pBMN.HIV-1MinWT.i.Zeocin or pBMN.HIV-1MinMut.i.Zeocin) and 3 µg of a vector expressing the Envelope glycoprotein of the Vesicular Stomatitis Virus (pCI-VSVg) by mixing the plasmids in 125 µl of FCS-free DMEM with 30 µg of Polyethylenimine (linear, MW 24000; Polysciences, Inc, Warrington, PA). Media (DMEM with 10% FCS, Pen-Strep, L-Glutamine) was replaced 24 hours post-transfection and viral supernatant was collected 48 hours after transfection and filtered through 0.45 micron PTFE filters (Pall Corporation). The supernatant was used to spin-infect naïve HEK293T or SupT1 cells in a six or twelve-well plate format. Briefly, viral supernatant was mixed with polybrene (5 µg/mL final concentrations) and added to the cells, the mixture plated in a six or twelve-well plate and spun at 1500 x g, 32^°^C for 80 min in a hanging bucket rotors centrifuge (Becton Dickinson). 24 hours post-infection, fresh media was added to cells.

### *Flow Cytometry and Sorting*

Cells were pelleted and incubated with mouse anti-FLAG (Sigma Aldrich, St. Louis, MO) and rabbit anti-HA (Cell Signaling, Beverly, MA) at 1:400 dilution for 20 minutes and then washed with PBS. Cells were then incubated with anti-mouse Alexa Fluor 488 and anti-rabbit Alexa Fluor 647 (Cell Signaling, Beverley, MA) antibodies at 1: 200 dilutions for 20 minutes and washed with PBS. Flow Cytometry and sorting were performed on a BD FACSCanto with 488nm and 633nm lasers and FACSAria with 488nm and 633nm lasers. Data was collected on FACSDiva 6.1.1 and analyzed using FlowJo 7.6.5.

### *Fluorescent Microscopy*

SupT1 cells were plated at 1x10^6^ cells per well on a twelve-well plate and treated with Furin Inhibitor I (EMD Millipore, Darmstadt, Germany) or DMSO control. Cells were collected and spun down for immunostaining in suspension. The cells were incubated with mouse anti-FLAG (Sigma) and rabbit anti-HA (cell signaling) at 1: 200 for one hour and then washed with PBS. The cells were then incubated with anti-mouse Alexa Fluor 555 and anti-rabbit Alexa Fluor 488 (Cell Signaling, Beverley, MA) for one hour and then washed with PBS. After antibody staining, cells were resuspended in 20 µl of fluorescence-preserving media and mounted on slides with cover slips. The imaging was performed using Zeiss Axio Observer D1 with an attached Zeiss MRc camera (Zeiss, Oberkochen, Germany) and analyzed using ImageJ software.
